# Towards sustainable processing of columbite group minerals: elucidating the relation between dielectric properties and physico-chemical transformations in the mineral phase

**DOI:** 10.1038/s41598-017-18272-3

**Published:** 2017-12-21

**Authors:** Sergio Sanchez-Segado, Tamara Monti, Juliano Katrib, Samuel Kingman, Chris Dodds, Animesh Jha

**Affiliations:** 1 School of Chemical and Process Engineering, University of Leeds, Leeds, LS2 9JT UK; 2 Microwave Process Engineering Research Group. Dept. of Chemical and Environmental Engineering, The University of Nottingham, Nottingham, NG7 2RD UK

## Abstract

Current methodologies for the extraction of tantalum and niobium pose a serious threat to human beings and the environment due to the use of hydrofluoric acid (HF). Niobium and tantalum metal powders and pentoxides are widely used for energy efficient devices and components. However, the current processing methods for niobium and tantalum metals and oxides are energy inefficient. This dichotomy between materials use for energy applications and their inefficient processing is the main motivation for exploring a new methodology for the extraction of these two oxides, investigating the microwave absorption properties of the reaction products formed during the alkali roasting of niobium-tantalum bearing minerals with sodium bicarbonate. The experimental findings from dielectric measurement at elevated temperatures demonstrate an exponential increase in the values of the dielectric properties as a result of the formation of NaNbO_3_-NaTaO_3_ solid solutions at temperatures above 700 °C. The investigation of the evolution of the dielectric properties during the roasting reaction is a key feature in underpinning the mechanism for designing a new microwave assisted high-temperature process for the selective separation of niobium and tantalum oxides from the remainder mineral crystalline lattice.

## Introduction

Niobium (Nb) and tantalum (Ta) are considered strategic materials due to their importance in high technology applications. These two elements are significant constituents for the manufacturing of super-alloys for the aerospace industry, high-strength low-alloy steel (HSLA) for construction, low temperature superconductor (LTS) wires for medical equipment (i.e. MRI and NMR), particle accelerators and nuclear fusion equipment. The oxides of niobium and tantalum are also used for manufacturing capacitors, soft-ferroelectric materials, and in the engineering of advanced bone implant materials^1,2^. Such applications in high-value product manufacturing have pushed the demand for these two materials, estimated at an annual increase rate of 4.7% for tantalum and 10% for niobium^1,2^. As a consequence, there is an encouraging trend for their recovery from scrap materials and low grade minerals.

Columbite group minerals (CGM) are the main source for the production of niobium and tantalum metals. The industrial treatment of CGM concentrates consists of the digestion of the concentrate using a mixture of sulphuric and hydrofluoric acid at temperatures between 250 and 300 °C to form niobium and tantalum fluorides, which are selectively separated by solvent extraction (SX) using an organic medium. The main drawbacks of the current technology are^3–5^:

- The leaching process is not selective and large amounts of impurities are co-dissolved with niobium and tantalum leading to huge costs associated with the regeneration and loss of fluorine.
- The organic extractants used in the SX processes are not optimized.
- The use of HF and organic solvents leads to potentially dangerous operations and serious environmental pollution.
- The process generates a substantial solid and liquid waste, which are difficult to dispose of because of their chemical reactivity.

In 1990 the production of 1 ton of niobium and tantalum oxides generated an average of 14 tons of solid waste of which 500 kg were radioactive^6^.The reduction of solid and liquid waste is still a major concern for the industry^5,6^. Current research has also focused on the reduction of the HF used and its replacement with gaseous mixtures based on chlorine gas^7–12^. However, the improvements achieved on a laboratory scale do not meet the sustainability criteria established in The World Summit on Sustainable Development held in Johannesburg on 2002^13^, which is why the industry sector continues to explore a sustainable approach for both utilizing mineral reserves and recycling. Recently, we reported^14–17^ the importance of alkali roasting and selective leaching technique with sodium or potassium salts as an alternative for the treatment of industrial minerals and wastes, namely ilmenite, bauxite and red mud, and critical metals tantalum, niobium and rare-earth metal oxide minerals.

## Dielectric heating of minerals

Recent studies from the Microwave Process Engineering group at the University of Nottingham demonstrate the economic viability of microwave heating at industrial scale for the beneficiation of copper minerlas^18,19^. Based on the results of mineral sorting trials, a crucial parameter that quantifies the storage of electromagnetic energy and the thermal conversion is the complex dielectric permittivity of the material as a function of the frequency, described by equation (1):

1 $$\varepsilon =\varepsilon ^{\prime} -j\varepsilon ^{\prime\prime} =\,{\varepsilon }_{o}\varepsilon {^{\prime} }_{r}-j{\varepsilon }_{o}\varepsilon {^{\prime\prime} }_{r}$$

where ε_0_ is the dielectric permittivity of the vacuum, ε′ and ε′′ are the real and imaginary parts of the complex dielectric permittivity, respectively, and ε′_r_ and ε′′_r_ are the real and imaginary part of the relative complex dielectric permittivity, respectively. The real part quantifies the storage of electromagnetic energy, whereas the thermal conversion is proportional to the imaginary part of the complex permittivity, also known as the loss factor^20^. During microwave heating, mineral constituents with higher values of ε′′ are selectively heated in comparison to the constituents with relatively lower loss factor values. However, differences in chemical composition, formation of reaction products and processing temperatures affect the dielectric properties which is why there is a need to understand the interaction of the CGM with microwave energy before considering microwave heating as an enabling tool for this process^21^.

In the context of engineering a novel technique for the extraction of niobium-tantalum oxides from columbite-tantalite minerals, we have reviewed the importance of dielectric properties of these two oxides for microwave processing, and on this basis we propose a combination of alkali roasting using microwave heating source as a first step in the conservation of energy and approaches for waste minimization. By characterising the dielectric properties in the microwave region, in future we will be able to make comparisons with the resistive and gas-fired heating methods. Also, the combination of microwave heating with alkali roasting will offer us an opportunity to design leaching process by minimizing the generation of hazardous waste.

## Methods

**Figure 1:**
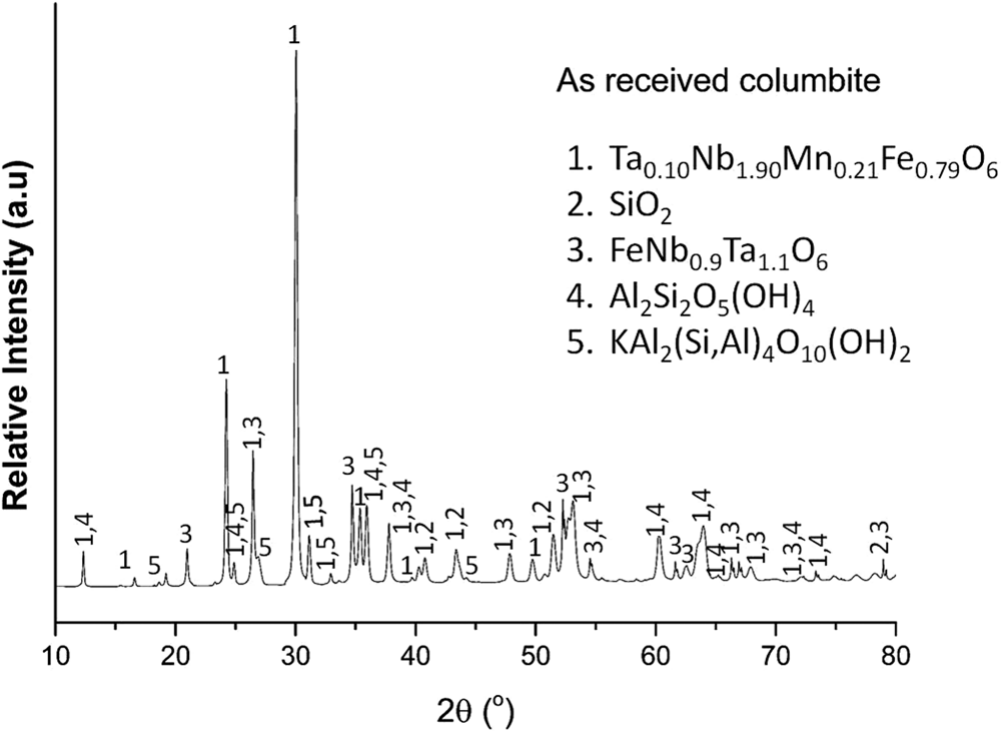
XRPD pattern of the as received columbite. The diffraction data compares well with ICDD refs. 04–012Ta_0.1_Nb_1.90_Mn_0.21_Fe_0.79_O_6_, 01-077-8310SiO_2_, 04-006-1584 Ta_1.1_Nb_0.9_FeO_6_, 00-029-1488 Al_2_Si_2_O_5_(OH)_4_, 00-058-2035 KAl_2_(Si,Al)_4_O_10_(OH)_2_.

The columbite concentrate from the Democratic Republic of Congo supplied by the Tantalum-Niobium International Study Centre was characterized using X-Ray powder diffraction (XRPD) with Cu-Kα radiation over an angle (2θ) range of 7 to 80°, X-Ray fluorescence (XRF) and scanning electron microscopy with energy dispersive X-ray analysis capabilities (SEM/EDX). Results are presented in the supplementary information Tables S1, S2 and Figure S1. The XRPD pattern in Fig. 1 shows that the major phases present in the mineral are ferro-columbite (n°1) and tapiolite (n°3), whereas muscovite (n°5), nacrite (n°4) and free silica (n°2) are identified as minor phases.

The dielectric properties of the columbite concentrate and its mixture 1:1 with sodium bicarbonate were characterized using the cavity perturbation method^22^ from room temperature to 1000 °C with increments of 100 °C between measurements. The powders were thoroughly mixed using mortar and pestle and held at each temperature for 10 minutes to allow the temperature to stabilise. The dielectric values were measured, using a vector network analyser (VNA) at a fixed frequency (2470 MHz), close to the allocated microwave heating frequency of 2450 MHz. Due to the short reaction times, a 1:1 ratio which corresponds to 1.5 times the stoichiometric amount of sodium bicarbonate, was employed to enhance the formation of the reaction products at lower temperatures. In order to relate the variation of the dielectric properties with the phase transformations occurring at each temperature, a direct comparison between the dielectric parameters and the thermo-gravimetric analysis (TGA) from room temperature to 1000 °C at a heating rate of 5 °C/min was also carried out. These experiments were complemented with isothermal roasting tests of the mixtures in air from 100° to 1000 °C with incremental steps of 100 °C in a CARBOLITE MoSi_2_ resistance furnace using recrystallized alumina crucibles. For isothermal tests, each sample was held at the target temperature for 15 minutes and then quenched in air for further examination using XRPD (Bruker D8 with X’Pert High Score plus software) and SEM/EDX (Carl Zeiss EVO MA15).

### Data availability

All data generated or analysed during this study are included in this article.

## Results and Discussion

**Figure 2:**
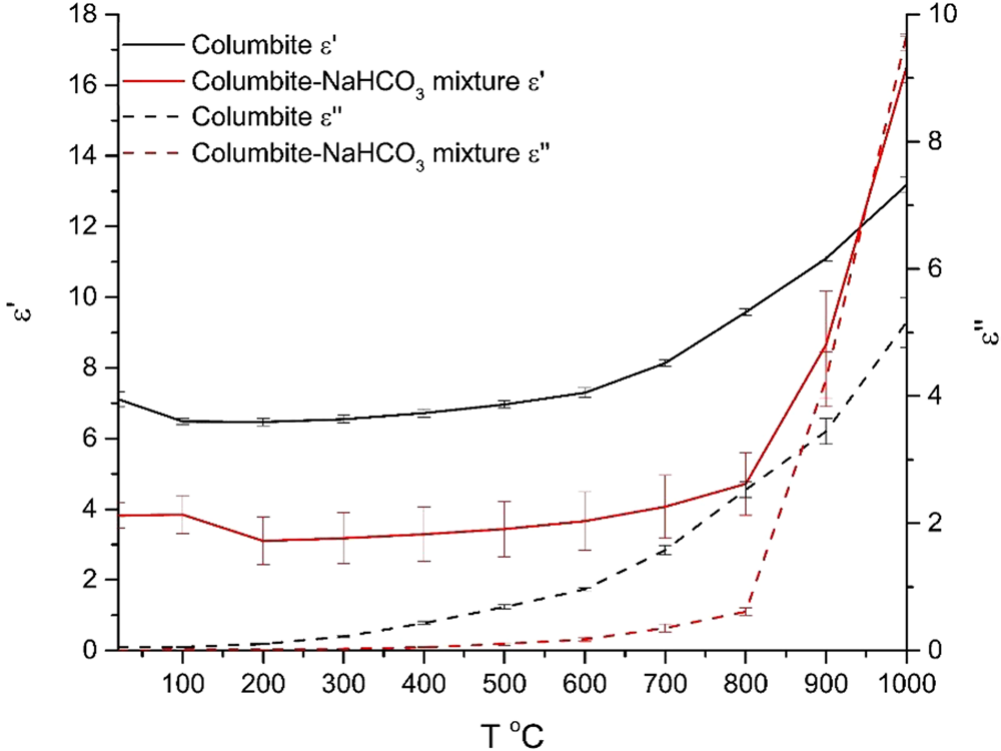
Comparison of the dielectric properties (average of 5 repetitions) of the columbite concentrate and its mixture with sodium bicarbonate at the industrial microwave frequency of 2470 MHz.

**Figure 3:**
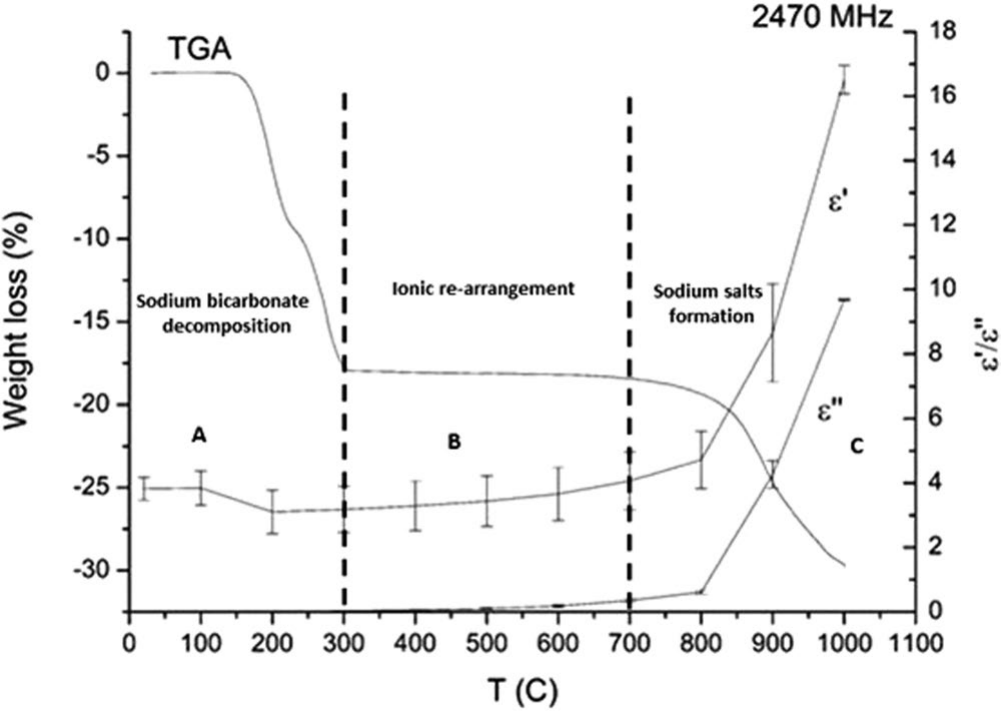
A correlation between the TGA weight loss and the dielectric properties ε′ and ε′′ with the temperature at the industrial microwave frequency of 2470 MHz.

From Fig. 2, it is evident that the conversion of electromagnetic energy into heat (represented by the ε′′ value) is dominated by the columbite concentrate, at least until 850C. After this temperature the value of ε′′ of the mixture grows exponentially until it reaches a value 1.6 times higher than the corresponding value for the columbite concentrate at 1000 °C as a consequence of the products formed after roasting reaction. In a supplementary micro-wave heating superimposed onto a thermogravimetric plot in Fig. 3, three main regions labelled from A to C can be identified according to the variations in the dielectric parameter values.

In Region A, the sample experienced a weight loss of roughly 17%. This decrease can be explained by the loss of moisture from the sample and the melting of NaHCO_3_ with simultaneous decomposition to Na_2_CO_3,_ carbon dioxide and steam in the range between 50° and 270 °C^23^. Additionally, the value of ε′ decreases to about 19% of its starting value from 100° to 200 °C. The conversion of NaHCO_3_ into Na_2_CO_3_ involves the breaking of the O-H dipole which mostly explains such a decrease in ε′. The values of the loss parameter ε′′ remain essentially constant up to 200 °C and then slightly increase from 200° to 300 °C. The increasing trend of the electrical conductivity of the Na_2_CO_3_ is probably the cause of the growing trend in ε′′ after 200 °C^24,25^. Other transformations observed within this temperature range include the incorporation of iron into the muscovite structure by forming a new mica phase, known as celadonite. This transformation is expected to occur due to the diffusion of iron ions from the columbite matrix to the muscovite inclusions^26^ (see Figures S2 and S3 and Table S3 in supplementary information). Both effects, the ionic mobility due to the liquid phase formation and the migration of the iron, contribute to increase the value of the dielectric loss from 200° to 300 °C.

**Figure 4:**
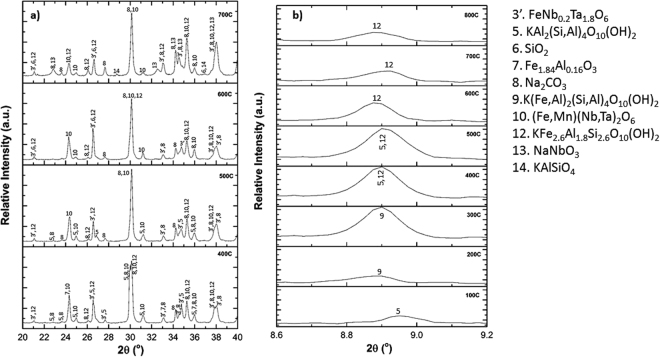
A comparison of XRPD patterns of samples roasted from 400 to 700 °C. (**a**) 2Ɵ; from 20 to 40° and (**b**) 2Ɵ; from 8.6 to 9.2°. The diffraction data compares well with ICDD refs.01–074 FeNb_0.2_Ta_1.8_ O_6_, 00-058-2034 KAl_2_(Si,Al)_4_O_10_(OH)_2_, 01-085-0865SiO_2_, 04-005-8669 Fe_1.84_Al_0.16_O_3_, 01-075-6816 Na_2_CO_3_, 00-033-0659 (Fe,Mn)(Nb,Ta)_2_O_6_, 04-017-1456 KFe_2.6_Al_1.8_Si_2.6_O_10_(OH)_2_, 04-012-8147 NaNbO_3_ and 04-012-8404 KAlSiO_4_. In Fig. 4b the changes in the diffraction intensity due to Fe^3+^ ion exchange between the original muscovite mineral (KAl_2_(Si,Al)_4_O_10_(OH)_2_) and the celadonite phase (K(Fe,Al)_2_(Si,Al)_4_O_10_(OH)_2_) are compared by examining the intensities of phases.

A slight monotonic increase in both the ε′ and ε′′ values recorded in Region B might be attributed to the process leading to the formation of NaNbO_3_ at 700 °C^17^, and the consequent release of Fe_2_O_3_, according to reactions (2)–(4), as verified in Fig. 4a.

2 $${{\rm{KFe}}}_{3}({{\rm{AlSi}}}_{3}{{\rm{O}}}_{10}){({\rm{OH}})}_{2}+3/4{{\rm{O}}}_{2({\rm{g}})}\to {{\rm{KAlSiO}}}_{4}+3/2{{\rm{Fe}}}_{2}{{\rm{O}}}_{3}+{{\rm{H}}}_{2}{{\rm{O}}}_{(g)}+2{{\rm{SiO}}}_{2}$$

3 $${{\rm{KFe}}}_{3}({{\rm{AlSi}}}_{3}{{\rm{O}}}_{10}){({\rm{OH}})}_{2}+{{\rm{Al}}}_{2}{{\rm{SiO}}}_{5}+3/4{{\rm{O}}}_{2({\rm{g}})}\to {{\rm{KAl}}}_{2}({{\rm{AlSi}}}_{3}{{\rm{O}}}_{10}){({\rm{OH}})}_{2}+1/2{{\rm{Fe}}}_{2}{{\rm{O}}}_{3}+{{\rm{SiO}}}_{2}$$

4 $$({\rm{Fe}},{\rm{Mn}}){({\rm{Nb}},{\rm{Ta}})}_{2}{{\rm{O}}}_{6}+{{\rm{NaHCO}}}_{3}\to ({\rm{Fe}},{\rm{Mn}}){{\rm{Ta}}}_{2}{{\rm{O}}}_{6}+{{\rm{NaNbO}}}_{3}+{{\rm{CO}}}_{2}({\rm{g}})+{{\rm{H}}}_{2}{{\rm{O}}}_{(g)}$$

**Figure 5:**
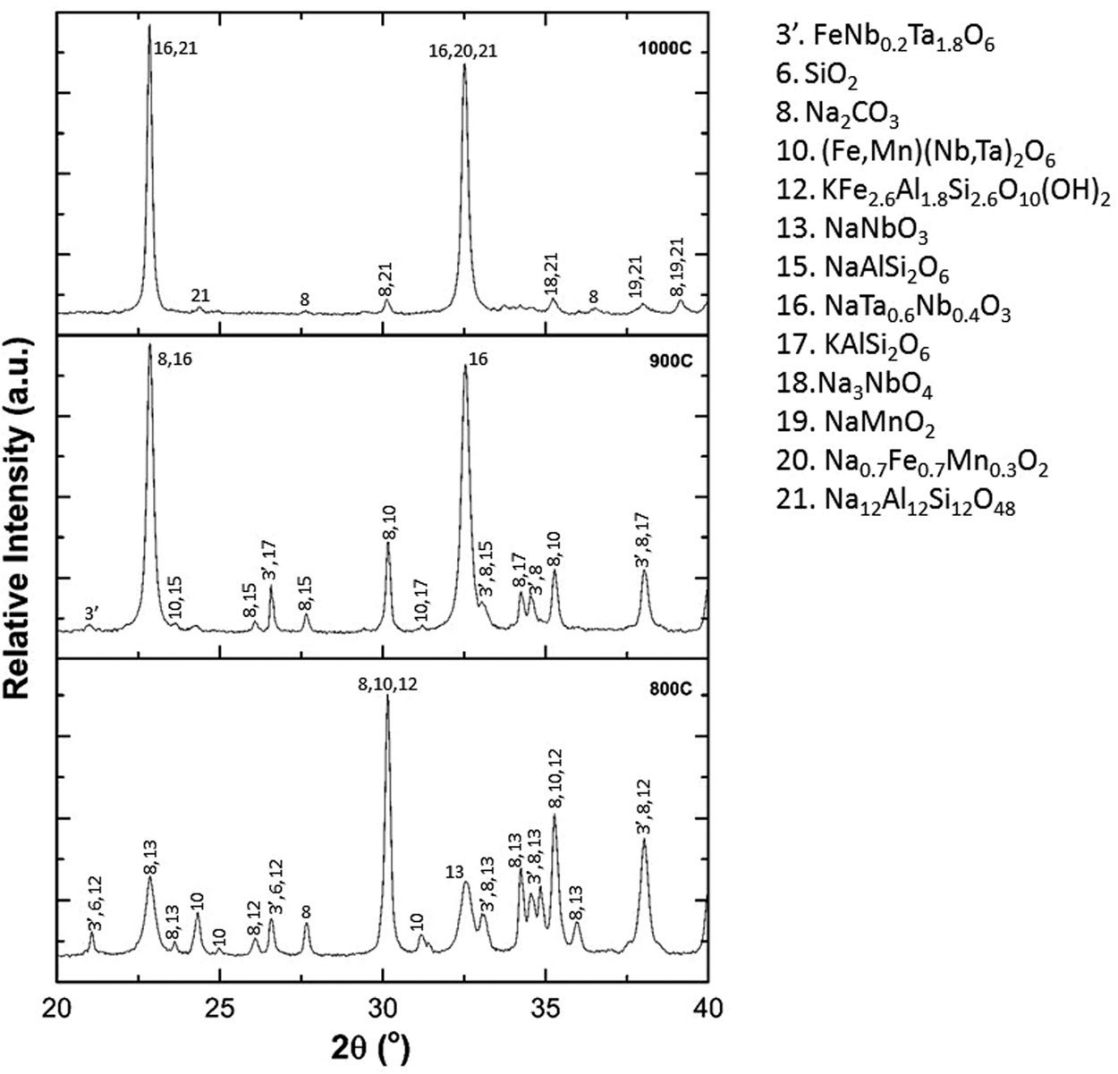
A comparison of the XRPD patterns of samples roasted from 800 to 1000 °C. Here the 2Ɵ; from 20 to 40° range has been selected, in which the diffraction data compares well with ICDD refs.01–074 FeNb_0.2_Ta_1.8_O_6_, 01-085-1780 SiO_2_, 04-011-4108 Na_2_CO_3_, 00-033-0659 (Fe,Mn)(Nb,Ta)_2_O_6_, 04-017-1456 KFe_2.6_Al_1.8_Si_2.6_O_10_(OH)_2_, 04-012-8147 NaNbO_3_, 01-082-9616 NaAlSi_2_O_6_, 04-006-6542 NaTa_0.6_Nb_0.4_O_3_, 00-031-0967 KAlSi_2_O_6_, 00-022-6542 Na_3_NbO_4_, 04-010-1809 NaMnO_2_, 00-053-0349 Na_0.7_Fe_0.7_Mn_0.3_O_2_ and 01-071-0370 Na_12_Al_12_Si_12_O_48_.

Iron (Fe^3+^), is incorporated into the muscovite structure (KAl_2_(Si,Al)_4_O_10_(OH)_2_) by forming a new family of mineral, known as celadonite (K(Fe,Al)_2_(Si,Al)_4_O_10_(OH)_2_) until 300 °C, which begins to segregate above 400 °C according to reactions (2) and (3). The analysis of the XRPD in the 2Ɵ range between 8.7 and 8.9°in Fig. 4b shows that the incorporation of iron in the structure results in a decrease of the 2θ values and an increase of the relative intensity of the peaks located within the range (see also Table 1)^27^. An apparent discrepancy in the measured 2θ values might be attributed to the overlap of the phases 5, 9 and 12, as can be deduced from the peak broadening in Fig. 4b. The SEM/EDX analysis are included in Figure S4 and Table S4 of the supplementary information. In the temperature range of region C (800°–900 °C), an exponential increase in the values of the dielectric properties was observed. This increase is attributed to the formation of niobium/tantalum sodium complexes observed in the XRPD pattern shown in Fig. 5, and in the supplementary SEM/EDX analysis included in Figure S5 and Table S5 of the supplementary information. The oxide NaTa_0.6_Nb_0.4_O_3_ has a perovskite structure with the general formula A^+^B^5+^O_3_, in which the B positions are occupied by the highly charged Nb^5+^ (0.78 Å) and Ta^5+^ (0.78 Å). Such a crystalline structure induces a spontaneous alignment of dipoles due to off-centre displacement of the B-site cations within their octahedral co-ordination, by imparting ferroelectric properties, which may be related with the exponential increase of the dielectric properties^28^.

**Table 1:** Comparison of the relative intensity and 2θ values for the peak located between 8.7 and 8.9° at different temperatures. ICDD ref. values KAl_2_(Si,Al)_4_O_10_(OH)_2_ = (8.889°, 58%), K(Fe,Al)_2_(Si,Al)_4_O_10_(OH)_2_ = (8.748°, 100%) and KFe_2.6_Al_1.8_Si_2.6_O_10_(OH)_2_ = (8.722°, 100%).

Temperature (°C)	2θ (°)	Relative Intensity (%)	Phases
100	8.9601	2.96	KAl_2_(Si,Al)_4_O_10_(OH)_2_
200	8.8855	3.80	K(Fe,Al)_2_(Si,Al)_4_O_10_(OH)_2_
300	8.9334	19.65	K(Fe,Al)_2_(Si,Al)_4_O_10_(OH)_2_
400	8.8988	17.24	KAl_2_(Si,Al)_4_O_10_(OH)_2_/ KFe_2.6_Al_1.8_Si_2.6_O_10_(OH)_2_
500	8.9099	14.13	KAl_2_(Si,Al)_4_O_10_(OH)_2_/ KFe_2.6_Al_1.8_Si_2.6_O_10_(OH)_2_
600	8.8845	10.37	KFe_2.6_Al_1.8_Si_2.6_O_10_(OH)_2_
700	8.9129	8.46	KFe_2.6_Al_1.8_Si_2.6_O_10_(OH)_2_
800	8.8915	6.43	KFe_2.6_Al_1.8_Si_2.6_O_10_(OH)_2_

The interacting microwave field distributes within the material under test, as apparent from the measured dielectric properties. The interacting field then is blocked by the resistivity (losses) encountered during its propagation within the sample. The depth at which the microwave power field decays to a factor 1/e (approx. 37%) of the value at the surface is conventionally assumed as the ‘penetration depth’ of the material and it is calculated by the following equation^29^:

5 $${D}_{p}=\frac{{\lambda }_{0}}{2\pi \sqrt{2\varepsilon \text{'}}}\frac{1}{\sqrt{[{\{1+{(\frac{\varepsilon ^{\prime\prime} }{\varepsilon ^{\prime} })}^{2}\}}^{0.5}-1]}}$$

where D_p_ is the penetration depth in meters, ε′ and ε′′ are the real and imaginary part of the permittivity of the medium, respectively, λ_0_ is the wavelength related to the operating frequency in free space. In the case of columbite-sodium bicarbonate mixture, the calculation of the penetration depth was performed at the different stages and temperatures of reaction and the results are reported in Table 2.

**Table 2:** Calculation of the penetration depth (m) for the roasting of the mixture columbite-sodium bicarbonate at 2470 MHz.

Temperature (°C)	*ε′*	*ε″*	δ (m)
20	3.82	0.008	4.723
100	3.84	0.010	3.788
200	3.10	0.011	3.094
300	3.18	0.024	1.436
400	3.29	0.052	0.674
500	3.44	0.104	0.345
600	3.66	0.177	0.209
700	4.07	0.355	0.110
800	4.72	0.612	0.069
900	8.66	4.263	0.014
1000	16.52	9.678	0.008

The differences in the penetration depth of the microwave field are important and will require careful consideration within the future development of this process in terms of energy uniformity applied to a batch or continuous process, especially at temperatures above 800 °C, for example the penetration depth of ~1.5 cm into the material at 900 °C. However, depending on the change in dielectric properties with frequency, it is expected that penetration depth will increase with decreasing frequency (for example at room temperature δ = 10.814 m at 910 MHz while it is δ = 4.723 m at 2470 MHz). Careful consideration of cavity/applicator geometry and material feed profile can address these limitations^29^.

## Conclusions

A correlation between the changes in the dielectric properties and the physico-chemical transformations in mineral phases occurring during the roasting of columbite with sodium bicarbonate has been investigated and analysed for the first time for engineering a microwave heating methodology for selective separation of tantalum and niobium oxide constituents as alkali complexes. The results show that there are three different regions which can be characterised by comparing the changes in the dielectric and thermogravimetric characterization, as shown in Fig. 3, in which the region A is dominated by the decomposition of sodium bicarbonate with a small decrease in the dielectric constant. In region B an ionic rearrangement of the columbite matrix occurs which is driven by the iron diffusion into the muscovite inclusions by forming celadonite type minerals. Further segregation of Fe_2_O_3_ from celadonite above 400 °C and the formation of NaNbO_3_ at 700 °C are the consequences of the slightly increase in the dielectrics observed in this region. The exponential increase in the dielectric properties observed in region C is attributed to the formation of sodium complexes especially to NaTa_0.6_Nb_0.4_O_3_.

The analysis of the penetration depth of the microwave heating shows that this parameter decreases at higher temperatures indicating the necessity for a careful selection of the reactor geometry in future, for the design of a process based on dielectric heating.

## Electronic supplementary material

Supplementary Information
