# Supplementary material for: Towards sustainable processing of columbite group minerals: elucidating the relation between dielectric properties and physico-chemical transformations in the mineral phase
Source: Sci Rep. 2017 Dec 21;7:18016. doi: 10.1038/s41598-017-18272-3 (PMC5740152; doi:10.1038/s41598-017-18272-3)
Supplement: Supplementary file 1 — Supplementary Information [file 41598_2017_18272_MOESM1_ESM.pdf]

Supporting information

Towards sustainable processing of columbite group minerals: elucidating the relation between dielectric properties and physico-chemical transformations in the mineral phase

Sergio Sanchez-Segado[a*], Tamara Monti[b], Juliano Katrib[b], Samuel Kingman[b], Chris Dodds[b] and Animesh Jha[a]

[a]School of Chemical and Process Engineering, University of Leeds LS2 9JT UK.

[b]Microwave Process Engineering Research Group. Dept. of Chemical and Environmental Engineering, The University of Nottingham NG7 2RD UK.

*Corresponding author: S.SanchezSegado@leeds.ac.uk

Columbite chemical composition (%w/w)									
**Ta_2O_5**	**Nb_2O_5**	**Fe_2O_3**	**MnO**	**Al_2O_3**	**SiO_2**	**K_2O**	**WO_3**	**SnO_2**	**Others**
39.00	31.10	16.30	4.39	1.27	2.60	2.43	0.54	0.85	1.52

Table S1. Chemical composition of the columbite concentrate analysed by XRF.

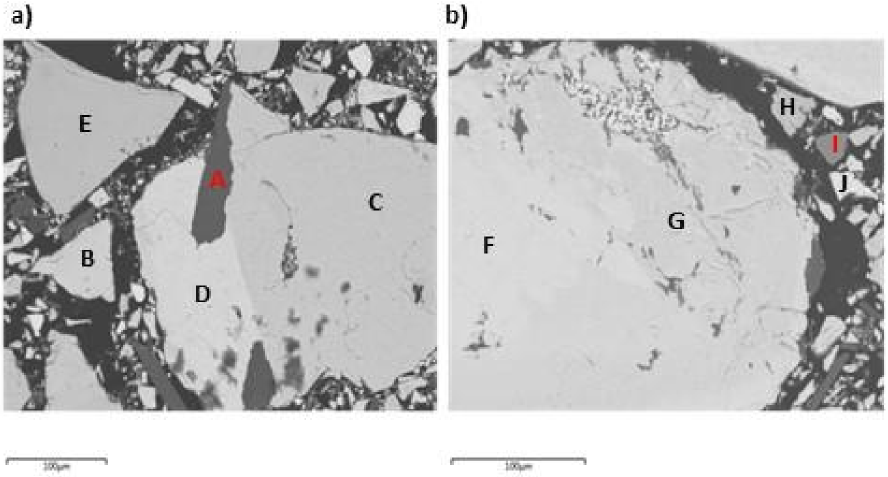

Figure S1a and S1b.Back-scattered electron images of columbite showing different phases (A-J).

**Area**	**Elemental Composition (%w/w)**								
	Nb	Ta	O	Fe	Mn	Al	Si	K	Phase
A	0.0	0.0	42.1	0.0	0.0	22.0	24.6	10.4	Muscovite
B	26.9	36.7	17.5	12.7	1.2	0.0	0.0	0.0	Ferro-tapiolite
C	34.1	27.9	19.4	0.0	14.4	0.0	0.0	0.0	Mangano-columbite
D	28.4	25.9	25.0	10.1	2.4	0.0	0.0	0.0	Ferro-columbite
E	44.1	17.2	20.2	0.5	16.4	0.0	0.0	0.0	Mangano-columbite
F	15.5	53.6	16.9	8.8	4.7	0.0	0.0	0.0	Ferro-tapiolite
G	27.8	39.4	17.4	12.4	2.6	0.0	0.0	0.0	Ferro-tantalite
H	43.3	15.8	20.8	12.2	3.7	0.0	0.0	0.0	Ferro-columbite
I	0.0	0.0	30.2	63.8	0.0	2.0	2.4	0.0	Hematite
J	0.0	70.7	15.4	12.5	0.0	0.0	0.0	0.0	Ferro-tapiolite

Table S2. EDX analysis of the areas A to J shown in figures S1a and S1b

The SEM images in Figures S1a and S1b, shown an oscillatory zonation (regions D, C, G and F) of the concentrate due to local fluctuations of the mineralization environment. Table S2, shows the EDX analysis of the columbite concentrate. The presence of hematite (area I), can be attributed to the oxidation of Fe^{2+} to Fe^{3+}, as a consequence of this, an extensive fractionation between Fe and Mn can be deducted during the mineral formation which explains the association of ferro-columbite- mangano-columbite and ferro-tapiolite-ferro-tantalite observed in the areas C-D and F-G respectively[1,2].

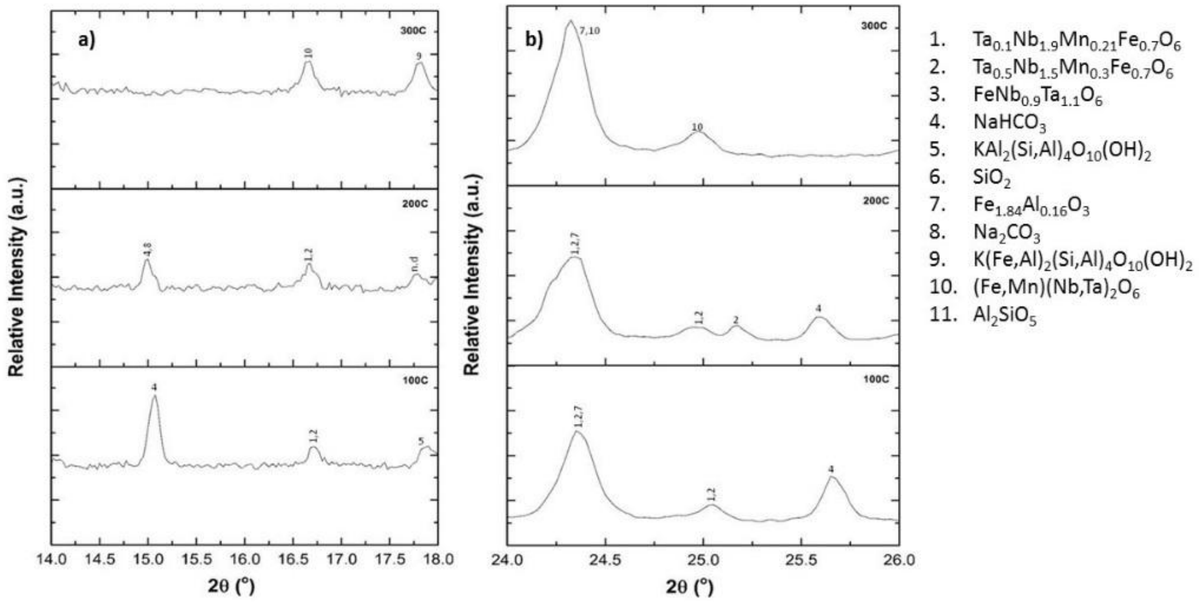

Figure S2a and S2b. XRPD patterns of samples roasted from 100 to 300°C a)2Ѳ from 14° to 18° c) 2Ѳ from 24 to 26°. The diffraction data compares well with ICDD refs.04-012-3523 $Ta_{0.1}Nb_{1.9}Mn_{0.21}Fe_{0.79}O_6$, 04-012-1553 $Ta_{0.5}Nb_{1.5}Mn_{0.3}Fe_{0.7}O_6$, 04-006-1584 $Ta_{1.1}Nb_{0.9}FeO_6$, 00-015-0700 $NaHCO_3$, 00-058-2034 $KAl_2(Si,Al)_4O_{10}(OH)_2$, 04-008-7819 SiO_2, 04-005-8669 $Fe_{1.84}Al_{0.16}O_3$, 01-075-6816 Na_2CO_3, 00-009-0439 $K(Fe,Al)_2(Si,Al)_4O_{10}(OH)_2$, 00-033-0659 $(Fe,Mn)(Nb,Ta)_2O_6$ and 01-070-7052 Al_2SiO_5

The XRPD patterns reported in Figure S2a show that $NaHCO_3$ and Na_2CO_3 coexists at 200°C with complete conversion into Na_2CO_3 at 300°C as indicated by the disappearance of the diffraction peak located at 15°, shown in Figure S2a.

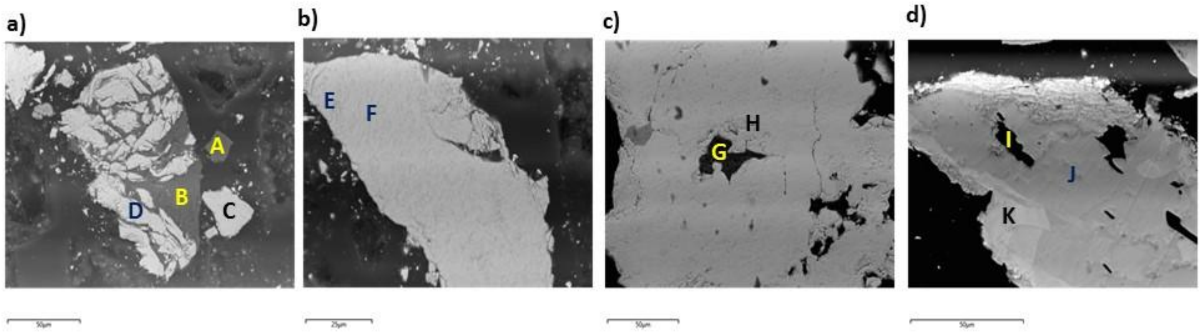

Figure S3. Back-scattered SEM images of the mixtures columbite-sodium bicarbonate (1:1) at 100 ºC a) and b), 200 ºC (c) and 300 ºC (c).

Area	Elemental Composition (%w/w)									
	Nb	Ta	O	Fe	Mn	Al	Si	K	Na	Phase
A	0.0	0.0	48.4	0.0	0.0	0.0	51.4	0.0	0.4	SiO_2
B	0.0	0.0	44.9	0.0	0.0	21.1	23.1	9.0	1.1	$KAl_2(Si,Al)_4O_{10}(OH)_2$
C	34.5	27.6	19.9	14.0	1.1	0.0	1.9	0.0	0.7	$Ta_{0.5}Nb_{1.5}Mn_{0.3}Fe_{0.7}O_6$
D	28.2	34.4	20.2	12.4	1.1	0.0	2.4	0.0	0.6	$Ta_{0.5}Nb_{1.5}Mn_{0.3}Fe_{0.7}O_6$
E	39.9	18.8	22.2	11.6	3.9	0.0	0.0	0.0	2.2	$Ta_{0.1}Nb_{1.9}Mn_{0.21}Fe_{0.79}O_6$
F	44.7	17.3	22.0	12.1	3.9	0.0	0.0	0.0	0.0	$Ta_{0.1}Nb_{1.9}Mn_{0.21}Fe_{0.79}O_6$
G	0.0	0.0	43.0	0.8	0.0	20.3	24.7	10.1	0.4	$K(Fe,Al)_2(Si,Al)_4O_{10}(OH)_2$
H	34.9	28.4	21.1	13.2	1.5	0.0	0.0	0.0	0.0	$Ta_{0.5}Nb_{1.5}Mn_{0.3}Fe_{0.7}O_6$
I	0.0	0.0	42.3	2.2	0.0	16.0	25.5	9.6	0.9	$K(Fe,Al)_2(Si,Al)_4O_{10}(OH)_2$
J	24.1	43.1	18.0	4.6	9.8	0.0	0.0	0.0	0.0	$(Fe,Mn)(Nb,Ta)_2O_6$

Table S3. EDX analysis of the areas A to J shown in figures S3a to S3d

In Table S3 the SEM/EDX analysis of the samples roasted from 100 to 300ºC show an increase in the iron content of the silicate phases (areas B, G and I). The columbite grains analysed in areas C-F and H show compositions which nearly falls between the

ones for $Ta_{0.1}Nb_{1.9}Mn_{0.21}Fe_{0.79}O_6$ and $Ta_{0.5}Nb_{1.5}Mn_{0.3}Fe_{0.7}O_6$pointing out the existence of solid solutions. Worthy of noting is the increase of the Mn proportion in area J which might be explained as a consequence of the iron incorporation into the silicate phases, enhancing the internal re-arrangement of the columbite matrix to form the $(Fe,Mn)(Nb,Ta)_2O_6$ phase founded in the XRPD patterns at 300ºC (Figure S2b).

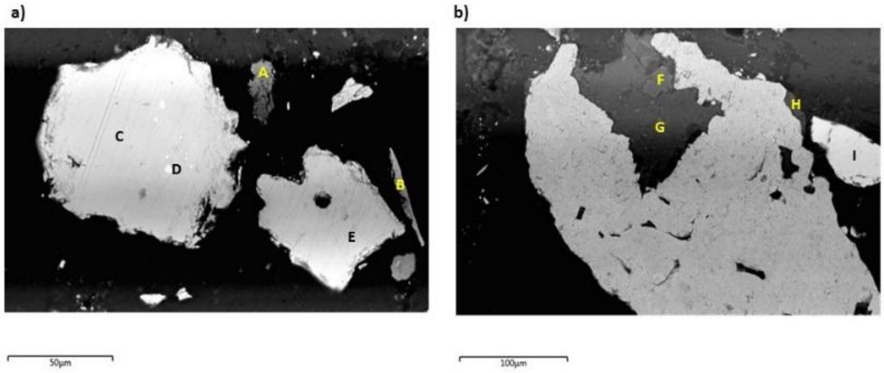

Figure S4. Back-scattered SEM images of the mixtures columbite-sodium bicarbonate (1:1) roasted at 600 ºC

Area	Elemental Composition (%w/w)									
	Nb	Ta	O	Fe	Mn	Al	Si	K	Na	Phase
A	0.0	0.0	19.6	6.6	70.5	0.0	0.0	0.0	2.7	MnO
B	42.0	19.8	20.5	0.8	16.6	0.0	0.0	0.0	0.4	$Mn(Nb,Ta)_2O_6$
C	4.2	66.7	14.7	12.1	0.0	0.0	0.0	0.0	0.0	$FeNb_{0.2}Ta_{1.8}O_6$
D	0.0	72.1	13.4	12.5	0.0	0.0	0.0	0.0	0.6	$FeTa_2O_6$
E	4.6	62.4	16.2	12.1	0.0	0.0	0.0	0.0	0.0	$FeNb_{0.2}Ta_{1.8}O_6$
F	0.0	0.0	44.6	0.3	0.0	0.0	55.1	0.0	0.0	SiO_2
G	0.0	0.0	33.3	12.4	0.0	23.2	27.4	2.7	0.6	$KFe_{2.6}Al_{1.8}Si_{2.6}O_{10}(OH)_2$
H	0.0	0.0	40.7	0.7	0.0	23.1	27.4	7.3	0.7	$KAl_2(Si,Al)_4O_{10}(OH)_2$
I	0.0	73.0	13.6	13.4	0.0	0.0	0.0	0.0	0.0	$FeTa_2O_6$

Table S4. EDX analysis of the areas A to I shown in figures S4a and S4b.

In areas F, G and H, it can be observed the co-existence of muscovite (H), annite (G) and silica (F) produced as a decomposition product. The incorporation of iron in muscovite to form annite changes the ratio (Mn/Fe+Mn) in columbite, as a result, the excess of manganese is segregated as MnO (area A). This internal re-arrangement promotes the decomposition of ferro-columbite in mangano-columbite (area B) and ferro-tapiolite (areas C, D, E and I).

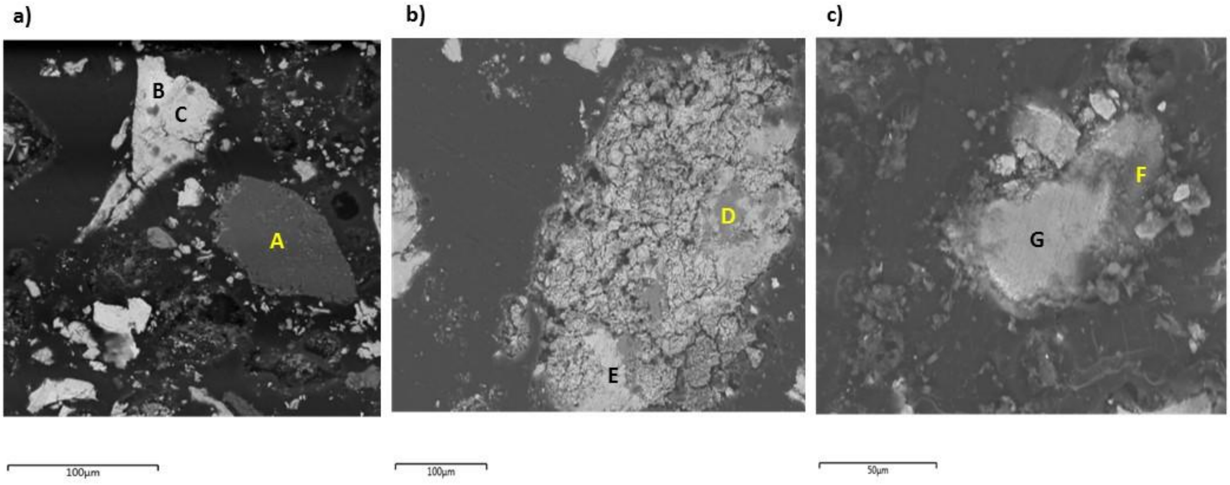

Figure S5. Back-scattered SEM images of the mixtures columbite-sodium bicarbonate (1:1) roasted at 900 ºC a) and 1000 ºC b) and c).

In the SEM images of Figure S5, it can be observed how Na^+ ions have diffused into the columbite matrix together with signals of melting and further recrystallization of the particles. Due to the short reaction time, it has been difficult to find completely segregated phases as can be observed in areas G and F in Table S5.

Area	Elemental Composition (%w/w)									
	Nb	Ta	O	Fe	Mn	Al	Si	K	Na	Phase
A	0.0	0.0	42.5	4.1	0.0	17.4	18.0	0.7	16.8	$Na_{12}Al_{12}Si_{12}O_{48}$
B	6.0	61.0	18.0	10.4	0.4	0.0	2.8	0.0	1.1	$FeNb_{0.2}Ta_{1.8}O_6$
C	5.4	49.9	19.8	10.7	0.0	0.0	4.3	0.0	8.4	$FeNb_{0.2}Ta_{1.8}O_6$
D	1.4	1.8	25.7	49.2	1.9	0.9	3.2	0.0	14.6	$NaFeO_2$
E	18.4	46.0	19.6	1.7	0.0	0.0	0.0	0.0	10.4	$NaTa_{0.6}Nb_{0.4}O_3$
F	27.6	13.9	29.1	6.2	2.5	0.0	1.9	0.0	18.5	$NaTa_{0.6}Nb_{0.4}O_3/Na_3NbO_4/Na_{0.7}Fe_{0.7}Mn_{0.3}O_2$ mixture
G	27.8	16.2	22.2	9.2	3.7	0.0	1.8	0.0	18.5	$NaTa_{0.6}Nb_{0.4}O_3/Na_3NbO_4/Na_{0.7}Fe_{0.7}Mn_{0.3}O_2$ mixture

Table S5. EDX analysis of the areas A to F shown in figures S5a, S5b and S5c.

References

1. Nova, M., Uher, P., Cerny, P., Siman, P. Compositional variations in ferrotapiolite-tantalite pairs from the beryl-columbite pegmatite at Moravany nad Va´ hom, Slovakia. *Miner. Petrol.* **69**, 295-306 (2000).
2. Cheng Wang, R.,Fontan, F.,Jin Xu, S., Ming Chen, X., Monchoux, P. The association of columbite, tantalite and tapiolite in the Suzhou granite, China. *Can. Mineral.* **35** 699-706 (1997).
